# Clinical Presentation, Genetics, and Laboratory Testing with Integrated Genetic Analysis of Molecular Mechanisms in Prader–Willi and Angelman Syndromes: A Review

**DOI:** 10.3390/ijms27031270

**Published:** 2026-01-27

**Authors:** Merlin G. Butler

**Affiliations:** Departments of Psychiatry, Behavioral Sciences and Pediatrics, University of Kansas Medical Center, Kansas City, KS 66160, USA; mbutler4@kumc.edu

**Keywords:** Prader–Willi and Angelman syndromes, review, clinical presentations, laboratory testing and genetic counseling, genomic imprinting, candidate or causative genes and defects, molecular genetic classes of chromosome 15q11-q13 region, integrated genetic analysis of predicted gene and protein interactions, clinical trials and treatment strategies

## Abstract

Prader–Willi (PWS) and Angelman (AS) syndromes were the first examples in humans with errors in genomic imprinting, usually from de novo 15q11-q13 deletions of different parent origin (paternal in PWS and maternal in AS). Dozens of genes and transcripts are found in the 15q11-q13 region, and may play a role in PWS, specifically paternally expressed *SNURF-SNRPN* and *MAGEL2* genes, while AS is due to the maternally expressed *UBE3A* gene. These three causative genes, including their encoding proteins, were targeted. This review article summarizes and illustrates the current understanding and cause of both PWS and AS using strategies to include the literature sources of key words and searchable web-based programs with databases for integrated gene and protein interactions, biological processes, and molecular mechanisms available for the two imprinting disorders. The *SNURF-SNRPN* gene is key in developing complex spliceosomal snRNP assemblies required for mRNA processing, cellular events, splicing, and binding required for detailed protein production and variation, neurodevelopment, immunodeficiency, and cell migration. The *MAGEL2* gene is involved with the regulation of retrograde transport and promotion of endosomal assembly, oxytocin and reproduction, as well as circadian rhythm, transcriptional activity control, and appetite. The *UBE3A* gene encodes a key enzyme for the ubiquitin protein degradation system, apoptosis, tumor suppression, cell adhesion, and targeting proteins for degradation, autophagy, signaling pathways, and circadian rhythm. PWS is characterized early with infantile hypotonia, a poor suck, and failure to thrive with hypogenitalism/hypogonadism. Later, growth and other hormone deficiencies, developmental delays, and behavioral problems are noted with hyperphagia and morbid obesity, if not externally controlled. AS is characterized by seizures, lack of speech, severe learning disabilities, inappropriate laughter, and ataxia. This review captures the clinical presentation, natural history, causes with genetics, mechanisms, and description of established laboratory testing for genetic confirmation of each disorder. Three separate searchable web-based programs and databases that included information from the updated literature and other sources were used to identify and examine integrated genetic findings with predicted gene and protein interactions, molecular mechanisms and functions, biological processes, pathways, and gene-disease associations for candidate or causative genes per disorder. The natural history, review of pathophysiology, clinical presentation, genetics, and genetic-phenotypic findings were described along with computational biology, molecular mechanisms, genetic testing approaches, and status for each disorder, management and treatment options, clinical trial experiences, and future strategies. Conclusions and limitations were discussed to improve understanding, clinical care, genetics, diagnostic protocols, therapeutic agents, and genetic counseling for those with these genomic imprinting disorders.

## 1. Introduction and Background

Prader–Willi (PWS) and Angelman (AS) syndromes are classical rare genetic disorders as the first examples in humans of errors in genomic imprinting. PWS is due to the absent expression of imprinted paternal alleles from chromosome15q11-q13 from the father and the absence of imprinted gene expression from the mother in AS [1,2,3,4,5,6,7,8,9,10,11,12,13,14,15,16,17,18,19,20,21]. A typical de novo deletion of this region is the most common defect in both disorders, depending on the parent of origin. In addition, maternally derived duplications of the 15q region may cause seizures, behavioral problems, and autism spectrum disorder, but are not diagnosed with PWS or AS. PWS is caused by more than one imprinted gene, but AS is due to the loss of a single paternally imprinted and maternally expressed *UBE3A* gene found in the 15q11-q13 region. About two dozen genes and transcripts are found in the 15q11-q13 region. Evidence suggests that two imprinted genes encoding proteins (*SNRPN* and *MAGEL2*), when disturbed by different genetic defects and grouped into three PWS molecular genetic classes, play key causative roles [14,15,16].

Angelman syndrome (AS) is an entirely different clinical disorder compared with PWS, but it is often referred to as a sister syndrome as they share similar defects of genomic imprinting, but opposite origin (maternal in AS and paternal in PWS). There are four recognized molecular genetic classes in AS, but both disorders share the typical 15q11-q13 deletion, with a different parent of origin. Individuals with AS are often not detected early by medical professionals, as few anomalies are noted at birth, and other clinical findings may not be reported until six months of age or later, unlike infants with PWS [5,7,11,22,23,24].

Both PWS and AS also share similar genetic defects depending on the parent of origin, with different phenotypes based on molecular genetic classes, mechanisms, and causes per patient [5,7,22,23,24]. Different treatment and surveillance approaches exist with separate genetic counseling risks for the two genomic imprinting syndromes [7,8,11,12,14,15,16,23]. Early accurate diagnosis and identification of the molecular genetic classes are essential for diagnosis, treatment, surveillance, and genetic counseling to inform and guide expectations and quality of life. Most cases of PWS or AS are thought to be sporadic [23,24].

The strategies for this review will focus on the current understanding of cause and diagnosis from literature and web-based sources, as well as natural history, clinical presentation, genetics, cytogenetics, molecular genetic testing, and protocols with experiences described for both classic genetic disorders. The documented literature sources, such as PUBMED, OMIM, and others, were searched using keywords related to these two imprinting disorders. Searchable validated web-based programs and databases were utilized throughout for both PWS and AS to identify gene and protein–protein interactive networks to characterize molecular functions, biological processes, pathways, cellular components, and gene–disease associations.

### 1.1. Clinical and Genetic Overview of Prader–Willi and Angelman Syndromes

#### 1.1.1. Clinical Presentation of Prader–Willi Syndrome

PWS is considered the most common genetic cause of life-threatening obesity in humans and affects an estimated 400,000 people worldwide [2], with approximately 200 babies born with PWS annually in the United States [1,2,15,16,17,24,25,26,27]. It is estimated at one in 20,000 live births; a comparable number is found for AS [14]. Infants with PWS present with a history of reduced fetal movement, severe hypotonia, poor suck, feeding difficulties, and failure to thrive. Hypogonadism/hypogenitalism with cryptorchidism are cardinal features. Short stature, small hands, and feet are due to growth and other hormone deficiencies with low muscle mass and reduced metabolism, requiring early assessment and treatment. Intellectual disability is mild (IQ 60 to 70), and speech problems are noted along with stubbornness, self-injury, outbursts, autistic features, temper tantrums, compulsions, and anxiety in childhood. Psychiatric problems often occur in adolescence and young adulthood, including mood instability, autism, and psychosis that correlate with molecular genetic classes [11,14,16,20,28,29,30,31]. Other clinical findings include sleep disturbances and daytime drowsiness [32,33], scoliosis, osteoporosis, temperature instability, decreased vomiting, and high pain threshold. Early characteristic craniofacial features include a narrow bifrontal diameter, blond to light brown hair with fair skin, strabismus, a small, upturned nose with a thin upper lip and downturned corners of the mouth, sticky saliva, and enamel hypoplasia [1,6,14,16,17,22,25,26,34].

Historically, PWS is divided into two clinical stages, with failure to thrive found in early infancy recognized as the first stage, while hyperphagia and onset of obesity are found in the second stage [1,14]. Nutritional phases include phase 0 with decreased fetal movement in utero, followed by phase 1 with hypotonia, failure to thrive, and difficulty eating; phase 2 with weight gain first noted at two years of age; and phase 3 with lack of satiety and food seeking with hyperphagia leading to obesity at about 7 years of age [35].

Hyperphagia continues into adulthood in PWS and can result in life-threatening obesity [16,26,36,37], including a risk of consuming inedible items or overeating excessively until stomach rupture, leading to death. The average age of death based on a 40-year mortality survey in PWS was approximately 30 years for both males and females [38]. Respiratory failure was the most common cause, accounting for 31% of all deaths, with 70% of deaths occurring in adulthood. Males showed an increased risk for presumed hyperphagia-related accidents/injuries and cardiopulmonary factors compared to females [38,39,40]. Early diagnosis and intervention should lower the risk of death.

#### 1.1.2. Genetics of Prader–Willi Syndrome

PWS was the first example in humans due to errors in genomic imprinting. Over 160 imprinted genes are currently recognized and classified in the human genome, accounting for about 1% of all genes [41]. They regulate fetal growth with influence on placental growth and function related to the nervous system [12,42]. The imprint can be tissue-specific, with DNA methylation impacting on epigenetic markers that repress gene activity based on parental origin and controlled by imprinting centers in the genome [43].

**Figure 1:**
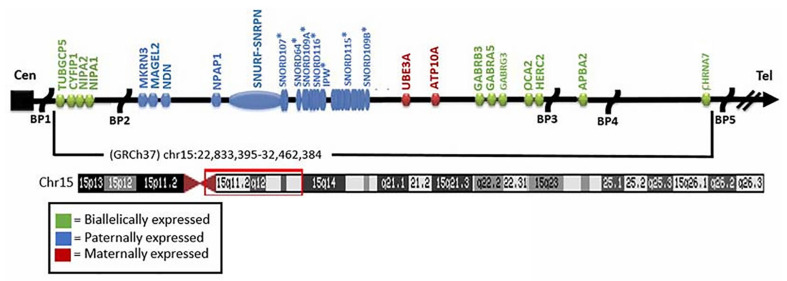
**Chromosome 15 ideogram.** Genes are shown in color and in linear order with five chromosome breakpoints (BP1-BP5). Breakpoints BP1, BP2 and BP3 are found in the 15q11-q13 region noted in the red frame on the ideogram and are common deletion sites for both PWS and AS. Biallelic (normal), paternal and maternal expressed genes are represented in different colors and the asterisk (*) in linear order represents non-coding RNA transcripts in the chromosome 15q11-q13 region.

Imprinted or nonimprinted genes and transcripts in the 15q11-q13 region in PWS or AS include *NIPA1*, *NIPA2*, *CYFIP1*, *TUBGCP5*, *MKRN3*, *MAGEL2*, *NDN*, *NIPAP1*, *SNURF-SNRPN*, non-coding *RNAs* (*SNORDs* including *SNORD116*, *SNORD115*, others), *UBE3A*, *ATP10A*, *GABRB3*, *GABRA5*, *GABRG3*, *OCA2,* and *HERC2* (see Figure 1). Several genes may influence clinical findings in PWS, but two imprinted genes (*SNRPN* and *MAGEL2*) are considered causative, while a single gene (*UBE3A*) causes AS. Two additional genes coding for proteases (*PCSK1* and *PCSK2*) not located in the 15q11-q13 region may be involved in PWS, as defects in prohormone convertase have been reported to impact key obesity-related genes such as *POMC* and its encoded list of peptides [44]. POMC is cleaved into separate peptides needed for appetite control, hypothalamic function, hormonal imbalances, and other biological mechanisms disturbed in PWS [15,45,46].

The *MKRN3*, *MAGEL2*, *NDN*, *NIPAP1,* and *SNURF-SNRPN* imprinted genes are expressed paternally and involved in PWS. For example, a defect of the *MAGEL2* gene causes Schaaf–Yang syndrome with features similarly seen in PWS [47]. Additionally, small deletions of the *SNRPN* and non-coding *SNORD116* transcript, which is not recognized as a gene, have been reported in patients with features seen in PWS, along with a single case of a de novo *SNRPN* variant from the father [16,48,49,50].

There are five recognized chromosome 15q breakpoints (two from the proximal region- BP1 and BP2, and three from the distal region- BP3, BP4, and BP5) with typical and atypical (smaller or larger) chromosome 15q deletions. The typical 15q11-q13 deletions involve proximal breakpoints BP1 or BP2 and the distal breakpoint BP3. The larger typical 15q11-q13 Type I deletion is about 6 Mb in size and involves BP1 and BP3, accounting for about 40% of the typical 15q11-q13 deletions. The smaller Type II deletion of BP2 and BP3 is about 5.5 Mb in size [15,16,23,51]. The region between BP1 and BP2 breakpoints include four highly conserved non-imprinted genes (*NIPA1*, *NIPA2*, *CYFIP1,* and *TUBGCP5*) (Figure 1).

PWS individuals with the larger Type I deletion often present with more learning and behavior problems, specifically maladaptive behaviors, compulsions, self-injury, and lower cognitive skills with seizures, compared to those with the smaller Type II deletion. The coefficient of determination for the deletion subtype alone explains up to 50% of the variation in the clinical parameters that were assessed when examining the expression of the four genes [29]. These observations may be attributed to the absence of the *NIPA1* and *NIPA2* genes found in the 15q11.2 BP1 and BP2 region that encode protein transporters for electrolytes, specifically magnesium, *CYFIP1*, and *TUBGCP5*, which contribute to developmental and behavioral changes [16,18,19,20,28,29,52,53]. Recent clinical studies from Brazil [54] and Italy [18] in PWS and chromosome 15 genetic defects showed variability in clinical findings, which may reflect differences in ethnic backgrounds, age at diagnosis, and treatment, including growth hormone.

Approximately 70% of individuals with PWS present with the typical 15q11-q13 deletion, while maternal disomy 15 accounts for about 25% of cases. About 5% have epimutations/microdeletions of the imprinting center, or other chromosome 15 rearrangements [48,49,50]. Imprinting center defects or unbalanced chromosome 15 translocations may have a 50% recurrence rate, e.g., father carries the imprinting defect, while the recurrence risks for other chromosome 15 defects would be less than 1% [14,51].

Clinical variation, specifically hypopigmentation, compulsions, and self-injury, is noted in those with typical 15q11-q13 deletions when compared with maternal disomy 15 [55]. Childhood-onset autism and psychosis in early adulthood occur more often with maternal disomy 15 [1,16,19,20,53] and increased risk of death from cardiopulmonary factors [38,39]. Furthermore, molecular genetic classes in PWS and growth hormone (GH) therapy impact intelligence and body mass index, and significantly higher IQ scores, particularly vocabulary, in growth hormone-treated pediatric-aged cohorts when compared with non-GH treatment [56]. There was a trend for stabilization of vocabulary scores with age in the GH-treated maternal disomy 15 subject group. Lower verbal IQ scores were found in the adult-aged cohort with the deletion compared with maternal disomy 15. However, no difference in body mass index was identified based on the molecular genetic classes.

#### 1.1.3. Clinical Presentation of Angelman Syndrome

Angelman syndrome (AS) was first reported by Dr. Angelman in 1965 [57] and further delineated [58]. Cardinal features include severe intellectual disability with marked delay in motor milestones, abnormal movement or balance with ataxia, seizures, and absent speech with fewer than six words. Frequent laughing is common but not apparently associated with happiness. Easy excitability is noted when engaged in certain activities with stimulation and uplifted arms with hand-flapping. Craniofacial features include micro-brachycephaly, recognized by 2 years of age with an open mouth, protruding tongue, drooling, and excessive chewing [59]. They often have blond hair and blue eyes with decreased skin pigmentation in those having the 15q11-q13 deletion of maternal origin, which involves the *OCA2* gene for pigment formation.

Deep-set eyes and a large mouth with a protruding tongue are found with widely spaced teeth and a prominent jaw [5,22,60]. Scoliosis, myopia with nystagmus, constipation, and obesity are noted in older children. Fascination with water is common, and drowning can be a cause of death [5,9,11,22,58]. Seizure activity is most severe at around four years of age but diminishes with time and may stop by 10 years. Seizures may vary from major motor to akinetic activity and are intractable with a characteristic EEG pattern with high-amplitude spikes and slow waves. Decreased sleep is reported in about 80% of cases, particularly between 2 and 6 years of age, along with abnormal sleep–wake patterns [9]. Increased sensitivity to heat with left-handed preference is also noted. Strong abilities are seen in manipulating electronics to assist in communication, but with challenging short attention spans [11,22]. Receptive abilities may be sufficient to understand simple commands. Most individuals with AS become toilet trained by day but are unable to live independently [3,5,22,58,61].

#### 1.1.4. Genetics of Angelman Syndrome

Several genetic mechanisms cause loss of function of the imprinted *UBE3A* gene and expression in the brain from the maternal allele only, including the same de novo interstitial 15q11-q13 deletion seen in PWS but of maternal origin. It is seen in approximately 70% of cases, while paternal disomy 15 accounts for about 5%. An imprinting center defect is seen in 3% to 5%, and a *UBE3A* variant in 10%. No identifiable molecular abnormality is found in about 10% of individuals clinically diagnosed with AS [11,22,61,62]. As seen in PWS, those with AS with cytogenetic deletions are more severely affected and diagnosed earlier [63], along with more developmental delays, seizures, and lack of pigmentation due to loss of the *OCA2* gene [1]. Variation in clinical presentations may trigger a referral for genetic services and diagnostic testing.

A recent AS cohort showed significantly decreased heights with the 15q11-q13 deletion, specifically during childhood and adolescence, as well as differences in body mass index, head circumference, and seizure activity [62]. Fujimoto et al. [64] also reported differences in walking independently related to chromosome 15 genetic defects. Those with the deletion had lower Bayley Scales of Infant and Toddler Development scores at baseline and a slower rate in gaining skills when compared to the nondeletion status [20,65].

Neurodevelopmental-behavioral problems are reported with deletions of the 15q11-q13 region between breakpoints BP1 and BP2 only or with the smaller involved 15q11.2 BP1-BP2 region (Burnside-Butler) syndrome [16,66,67,68]. This region is also deleted with PWS or AS having the larger Type I deletion and considered as a susceptibility locus with reduced penetrance and variable expressivity for learning, behavior, and motor control issues, dyslexia, hypotonia, compulsions, and cardiac problems [16,22,52,66,67,69]. About 90% of patients carrying this small deletion involving only four genes have developmental delays, intellectual disability, and other clinical findings [22].

To further assess the 15q11.2 BP1-BP2 deletion and role in neurodevelopment, commercial laboratory testing was undertaken by Ho and others [70] with ultra-high resolution chromosome microarrays on 10,000 patients presenting with neurodevelopmental disorders. Their study pinpointed genetic causes that are critical for clinical care and genetic counseling. Overall, a 28% diagnostic yield was found, and 85 genetic defects were identified, 9% had 15q11.2 BP1-BP2 deletions, followed by 16p11.2 deletions or duplications at 5% each. This proximal 15q11.2 deletion, genes, and encoded proteins will impact both PWS and AS.

## 2. Review of Data Strategy

To review and characterize information about PWS and AS, comprehensive searches were undertaken from the published literature sources and interactive web-based programs with databases focusing on *SNRPN*, *MAGEL2,* and *UBE3A* genes and their encoded proteins. Keywords related to these imprinting disorders were searched using PUBMED (https://pubmed.ncbi.nlm.nih.gov, accessed on 25 May 2025), OMIM (http://www.omim.org, accessed on 25 May 2025), Gene Reviews (http://www.genereviews.org, accessed on 25 May 2025), and Gene Cards (http://www.genecards.org, accessed on 25 May 2025). In addition, three publicly available web-based programs and databases were searched, including STRING (www.string-db.org, accessed on 25 May 2025) [71]; BioGRID (https://theBioGRID.org, accessed on 25 May 2025); and PathwayCommons (www.PathwayCommons.org, accessed on 25 May 2025). This query in humans (*Homo sapiens*) was carried out from 1 January 2025 to 31 August 2025 as access dates.

The STRING web-based program and database were used to identify predicted protein–protein interactions, characterized genetic mechanisms, molecular functions, and disease pathology with disturbances. The interactions represent both direct (physical) and indirect (functional) protein associations, derived from genomic context predictions, high-throughput laboratory experiments, automated text mining from the literature sources, and other databases with conserved co-expression patterns of genes. It provides a statistical approach for analyzing genes and their encoded proteins with four separate criteria. These include the ***Count-in-network*** function, which indicates how many proteins are in the visualized network and annotated with a particular term. ***Strength*** describes the size of the enrichment effect in a visualized network relative to the number of proteins expected to be annotated in a random network of the same size. ***False discovery rate* (*FDR*)** examines the significance of enrichment to conceptualize the rate of type I (false-positive) errors in the null hypothesis with reported *p*-values corrected for multiple testing. ***Signal*** is defined as a weighted harmonic mean between the observed/expected ratio and -log (FDR), meant to balance the metrics of larger and smaller terms for ordering of enriched terms.

Biological General Repository for Interaction Datasets (BioGRID) is a separate searchable program for protein–protein interactions from curated archive data for dissemination. It is used to study genetic and protein–protein functional interactions with related proteins assembled and updated monthly from relevant sources to include Gene Ontology biological processes, molecular functions, and cellular components.

Pathway Commons is the third web-based program used to access and identify gene-gene regulatory networks, functional interactions, and pathways enriched in gene expression and binding data among the interactive or shared processes. The three web-based programs collectively focused on protein–protein and/or gene–gene associations, functional mechanisms with related protein networks for the top biological processes, molecular functions, cellular components, pathways, and disease–gene associations.

## 3. Results and Discussion

Prader–Willi and Angelman syndromes are classified as sister disorders as examples of genomic imprinting. There are dozens of genes/transcripts within this region, with many being imprinted and dependent on the parent of origin. The clinical and genetic overview of the two disorders and the status of three presumed causative imprinted genes (*SNRPN* and *MAGEL2* in PWS and *UBE3A* in AS) are described and illustrated. Three separate web-based programs and databases were used to review the role of these causative genes and predicted genetic or protein–protein interactions, biological processes, functional mechanisms, and enrichment from genetic and protein networks.

### 3.1. Key Imprinted Genes and Computational Biology in Prader–Willi and Angelman Syndromes

#### 3.1.1. Prader–Willi Syndrome

Prader–Willi syndrome is impacted by the expression of several genes in the 15q11-q13 region. *SNRPN* and *MAGEL2* are potentially key imprinted causative genes. Imprinted genes have features in common with more than 80% localized into 16 clusters in the genome, with each containing at least two imprinted genes [14,72,73,74]. Imprinting factors regulate multiple genes, with each cluster containing a DNA sequence that is methylated in oogenesis, leading to a maternal imprint and paternally expressed genes and methylated in spermatogenesis for a paternal imprint and maternal expression. Nearly all clusters express a long non-coding RNA such as *SNURF-SNRPN*. DNA methylation causes an epigenetic mark that represses transcription in promoter regions that are controlled through an imprinting controlling element referred to as an imprinting center (IC). DNA methylases remove the DNA methylation pattern in the germline to reset the imprint. Histone modifications also contribute [14,73]. Compared with other recognized imprinting centers, such as Igf2r (insulin-like growth factor 2 receptor), the PWS/AS imprint is more complicated due to the large number of genes in the cluster (at least seven) with mixed parental patterns. Many imprinted genes are paternally expressed in PWS but only two genes (*UBE3A* and *ATP10A*) in AS [14,73].

##### Key Imprinted Genes in Prader–Willi Syndrome

*SNRPN*

The *SNRPN* (small nuclear ribonucleoprotein polypeptide N) gene consists of at least 8 coding exons and 11 non-coding upstream exons with three major transcriptional start sites found in exons 1, U1A, and U1B. *SNRPN* is imprinted, and the transcript encodes a polypeptide of a small nuclear ribonucleoprotein complex that belongs to the snRNP SMB/SMN family. It plays a role in pre-mRNA processing and tissue-specific alternative splicing that impacts protein production. The protein arises from a bicistronic transcript that encodes two proteins identified as SNRPN and SNRPN upstream reading frame (SNURF) from one mRNA. This coding *SNURF-SNRPN* mRNA contains two open-reading frames, *SNURF* and *SNRPN,* that both encode nuclear proteins [20,72,73,74]. This complex gene harbors numerous noncoding RNAs in a large (>440,000 bp) 3′ untranslated region (UTR), among them at least six classes of C/D box small nucleolar RNAs (snoRNAs) and the IPW (imprinted in Prader–Willi). The *SNRPN* gene is key in developing complex spliceosomal snRNP assemblies required for mRNA splicing and detailed protein production.

The promoter region of the *SNURF-SNRPN* gene overlaps with the imprinting center necessary for methylation of the parental allele. Multiple transcription initiation sites have been identified with extensive alternative splicing that occurs in the 5′ untranslated regions, identified as an imprinting center with alterations or deletions of this paternally expressed region responsible for PWS.

*MAGEL2*

*MAGEL2* is an imprinted paternally expressed gene from the melanoma antigen gene family member L2 that interacts with multiple genes, both imprinted and not imprinted. It encodes MAGE-like protein 2 that enhances E3 ubiquitin-protein ligase activity of a RING-type zinc finger. This occurs possibly through recruitment and stabilization of the Ubl-conjugating enzyme (E2) at the E3-substrate complex. It is involved in protein degradation and apoptosis by acting as a regulator of retrograde transport. It also interacts with VPS35 (lysosomal degradation) and TRIM27 (inhibits CD4 T-cell activation), noted as the MAGEL2-USP7-TRIM27 (MUST) complex needed for promotion of endosomal F-actin assembly and tissue-specific alternative RNA processing. MAGEL2 is also involved with the regulation of the circadian clock with rhythmic processing and repressing transcriptional activity affecting reproduction, sexual development, hormones (e.g., oxytocin), and eating behavior. Defects of this gene also cause Schaaf–Yang syndrome with overlapping features seen in PWS.

##### Computational Biology in Prader–Willi Syndrome

*SNRPN*

**Figure 2:**
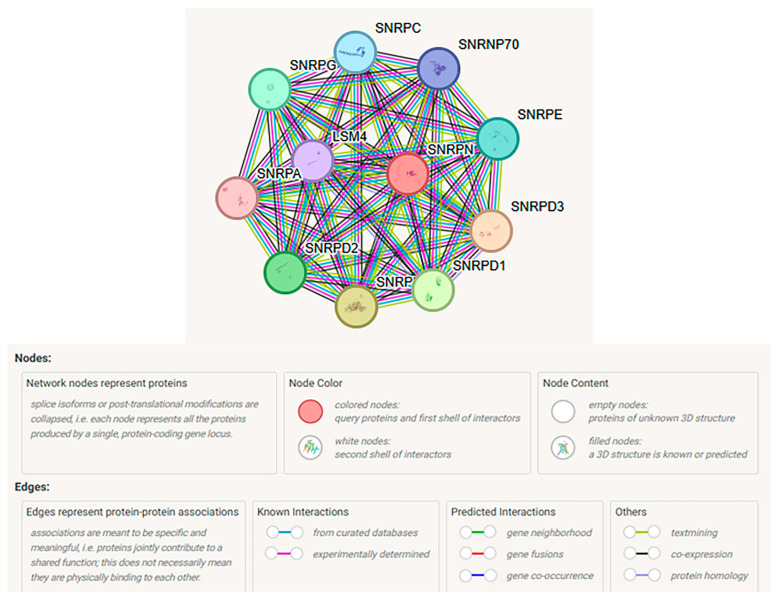
STRING protein–protein interaction network involving SNRPN gene transcript with functional interactions showing 10 shared protein nodes with 55 interactive edges (STRING Consortium 2023) with color coding illustrated and further described by Szklarczyk et al. [71]. Network nodes represent proteins with splice isoforms or post-translational modifications collapsed into each node for all proteins produced by a single protein-coding gene. Edges represent protein–protein associations that are considered specific and meaningful, i.e., proteins jointly contributing to a shared function. The different colored lines and nodes represent the known and predicted interactions and query proteins in the network.

The STRING computer web-based program was used, as explained in the Review Data Strategy section, to search SNRPN and the top ten significantly associated proteins. The search found several related genes, *SNRPD3*, *SNRPF*, *SNRPD1*, *SNRPD2*, *SNRPG*, *SNRPE*, *SNRPC*, *SNRNP70*, *LSM4*, and *SNRPA*, with their encoded proteins. Each node represents all proteins that were produced, including isoforms for every single protein-coding gene, and 55 protein edges representing both direct predicted functional roles and physical protein associations, processing, or interaction (see Figure 2).

The top 10 significantly associated proteins identified, and their 55 interactive edges were based on groups of genes frequently found in each other’s genomic neighborhood, gene fusions and gene co-occurrences supported by text-mining, co-expression and protein homology. The STRING network found more interactions than would be expected from a random set of proteins of the same size and degree of distribution drawn from the genome indicating that these proteins are at least partially biologically connected as a group. This analysis lends important evidence for spliceosome formation and spliceosomal snRNP assembly required for mRNA processing, including histones, splicing, and binding with protein production and specific isoform development. Descriptive functional findings of each of the 10 interrelated genes with SNRPN and their related encoded proteins are described in Table 1. This table shows involvement of the normal U2-type pre-spliceosomal assembly, mRNA splicing, and the DNA methylation status, and when disturbed, impacts critical protein production in PWS.

All 10 associated protein nodes were involved in mRNA splicing and RNA binding within the cellular spliceosomal snRNP complex, as shown in Table 1. Eight nodes were involved with spliceosomal assembly, and six nodes for DNA methylation impacting the activity of imprinted genes, also shown in Table 1. The top biological process was spliceosomal snRNP assembly, and eight nodes were recognized—SNRPC; SNRPG; LSM4; SNRPD2; SNRPF; SNRPD1; SNRPD3; and SNRPE. The top molecular function was snRNA binding with five recognized nodes—(SNRPC; SNRNP70; SNRPA; LSM4; and SNRPD3). Three nodes (SNRPC, LSM4, and SNRPD3) were common in the top biological process and molecular function identified for the *SNRPN* gene. The biological processes and functions associated with the *SNRPN* gene and related proteins are shown in Table 2.

*MAGEL2*

**Figure 3:**
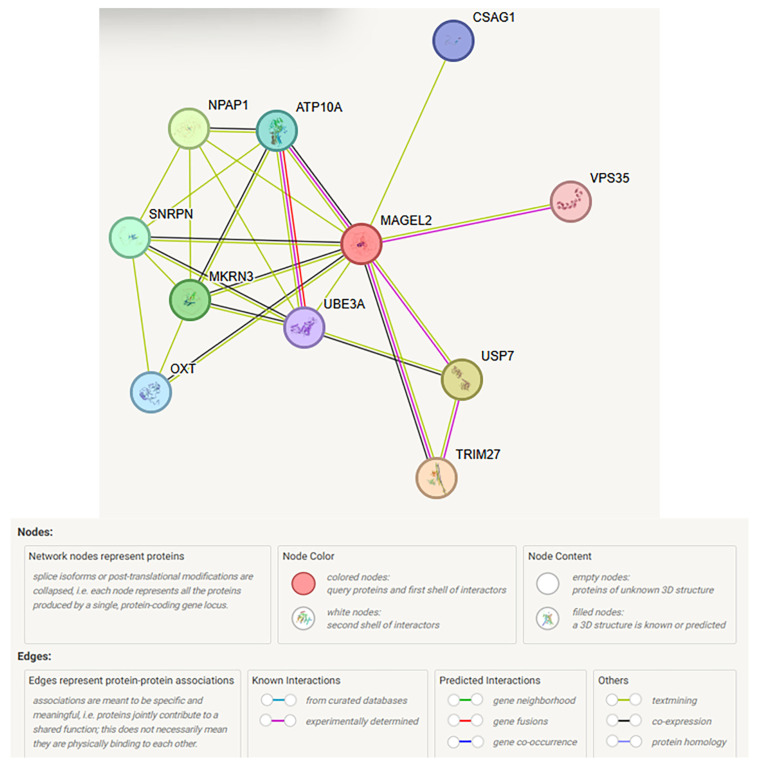
STRING protein–protein interaction network involving the MAGEL2 gene, with functional interactions showing 10 protein nodes for MAGEL2 with 24 interactive protein edges representing different functions. The predicted functional associations included biological processes and molecular functions [71].

The STRING website search identified the top 10 significantly interactive proteins with MAGEL2, including TRIM27, USP7, MKRN3, SNRPN, ATP10A, OXT, CSAG1, UBE3A, NPAP1, and VPS35. There were 10 associated protein nodes, each representing all proteins produced, including isoforms for every single protein-coding gene. The 24 edges indicate both direct predicted functional roles and physical protein associations, processing or interactions, and descriptions (see Table 3 and Table 4; Figure 3).

The proposed functions of *MAGEL2* and ten associated genes, which encode interactive proteins, lend evidence for transcriptional activity and regulation for biological or circadian rhythms, ubiquitin ligase activity for protein degradation, and apoptosis. Cellular organelle function and hormone production are also involved. Three nodes (MAGEL2, UBE3A, and USP7) were recognized as annotated keywords for biological rhythms, and five nodes (MAGEL2, MKRN1, UBE3A, USP7, and TRIM27) for the Ubl conjugation pathway, with three nodes in common (USP7, UBE3A, and MAGEL2) targeting proteins or lipids to regulate their activity, stability, localization, or interactions. For disease–gene association, four nodes were found, including SNRPN, MKRN3, MAGEL2, and UBE3A, while Schaaf–Yang syndrome showed two nodes (MAGEL2 and TRIM27). Angelman syndrome showed two nodes, as well (SNRPN and UBE3A). Human phenotype classification showed premature pubarche (five nodes), poor suck (three nodes), central sleep apnea (three nodes), early onset of sexual maturation (five nodes), and polyphagia (three nodes) when searching MAGEL2 with the STRING web-based program.

The summary of human phenotypes shown in Table 4 is based on the connection between *MAGEL2* gene defects and overlapping features seen in PWS and Schaaf–Yang syndromes. Loss of *MAGEL2* gene function and relationship to PWS includes hypothalamic neuroendocrine dysfunction, cellular defects with decreased secretory granule and neuropeptide production, as well as abnormal reproduction and circadian rhythms seen in both PWS and Schaaf–Yang syndrome [75].

##### Biological General Repository for Interaction Datasets (BioGRID)

BioGRID is a separate, searchable web-based program and dataset used for protein–protein functional interactions. The results from this search of SNRPN and MAGEL2 are shown in Table 5.

##### Pathway Commons Web-Based Program

**Figure 4:**
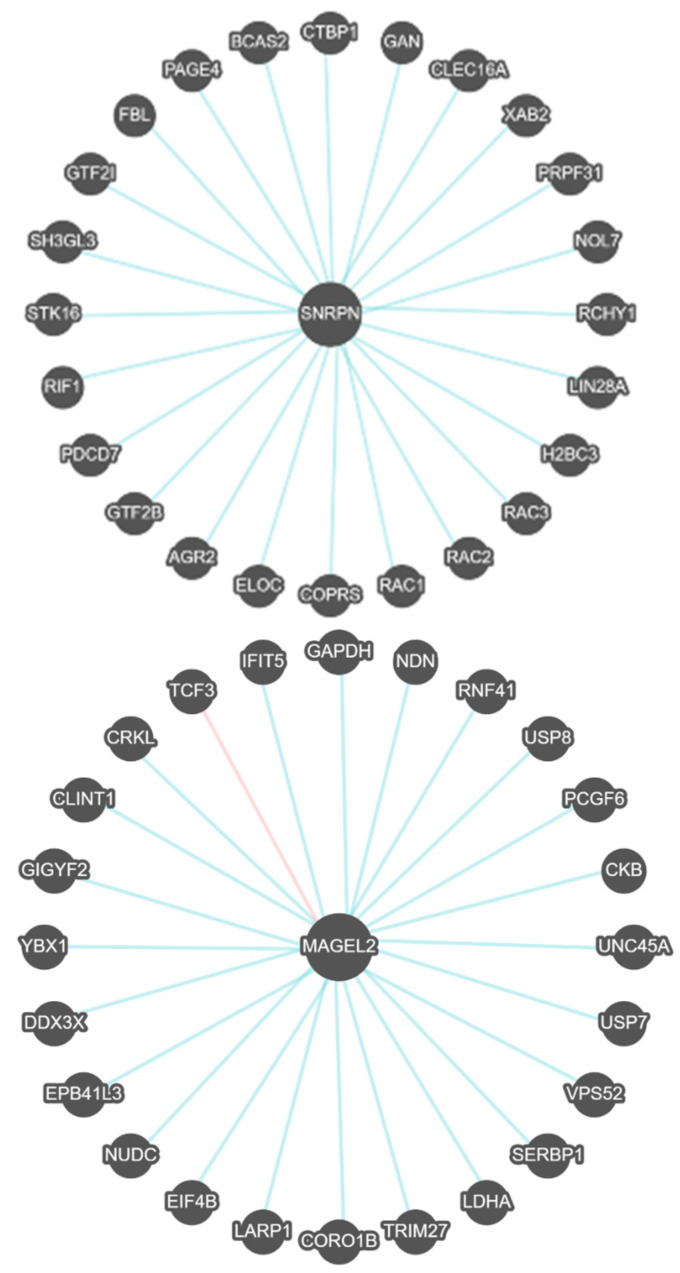
*SNRPN* and *MAGEL2* gene–gene functional interactions represented by binding (blue lines) and co-expression (red lines) for Prader–Willi syndrome.

Pathways Commons is a separate searchable program that focuses on gene–gene regulatory networks, functional interactions, and pathways enriched for related gene expression patterns and binding among interactive or shared processes. Figure 4 shows the gene–gene interactions for both *SNRPN* and *MAGEL2* genes in PWS.

In Figure 4, the ***SNRPN*** gene interacts with 24 genes and three members of the RAC gene family (*RAC1*, *RAC2*, *RAC3*), which code for GTPases belonging to the RAS superfamily of small GTP-binding proteins that regulate cellular events and growth, cytoskeletal reorganization, and protein kinase activation involving neurodevelopment and immunodeficiency. Other genes impact pre-mRNA and mRNA splicing factors (*BCAS2*, *PDCD7*, *XAB2*); general transcription factors (*GTF2I*, *GTF2B*) and elongation (*ELOC*); protein autophosphorylation (*STK16*); reproductive tissue expression factors (*PAGE4*); endoplasmic reticulum protein and a role in cell migration and function (*AGR2*); core histone protein H2B (*H2BC3*); fibrillarin or component of snRNP particle (*FBL*); early endosome membrane development (*SH3GLK3*); replication timing regulatory factor 1,DNA repair and telomere location (*RIF1*); nucleosome structure; posttranscriptional regulation and timing (LIN28A); C-terminal binding protein (*CTBP1*); ubiquitin ligase activity, ubiquitination and proteasomal degradation of targeted proteins impacting neurofilament architecture (*RCHY1*, *GAN*); regulator of mitophagy (*CLEC16A*); spliceosome complex (*PRPF31*); and component of ribosomal subunit (*NOL7*) (OMIM www.omim.org, accessed on 25 May 2025) (GeneCards www.genecards.org, accessed on 25 May 2025).

In Figure 4, the ***MAGEL2*** gene interacts with 24 genes and two genes (*USP7* and *USP8*) belonging to the ubiquitin-specific processing protease family of proteins to regulate the morphology of the endosome by ubiquitination of proteins and membrane trafficking required for the cell to enter the S phase of the cell cycle. Other genes and their encoded proteins impact carbohydrate metabolism and energy (*GAPDH*); transcription repressors (PCGF6); retrograde transport from endosome to trans-Golgi network (*VPS52*); RNA and DNA binding involved in defense to virus located in several cellular components (*IFIT5*); an E protein class that activate transcription by binding to regulatory E-box sequences on target genes (*TCF3*); encodes protein kinase containing SH2 to activate the RAS and JUN kinase signaling pathways (*CRKL*); trafficking between Golgi and endosomes (*CLINT1*); regulation of tyrosine kinase receptor signaling (*GIFYF2*); cold shock protein with nucleic acid binding properties and a role in microRNA processing (*YBX1*); transcriptional regulation, pre-mRNA splicing and export (*DDX3X*); cytoskeletal anchor protein involved in nervous system (*EPB41L3*); spindle formation for cell division (*NUDC*); RNA binding activity involved in eukaryotic translation initiation factor 4F (*EIF4B*); RNA and eukaryotic initiation factor binding and post- transcription and involved in male germ cell differentiation (*TRIM27*); SUMO, mRNA 3′-UTR and ribosome and binding activities (*SERBP1*); cytoplasmic enzyme involved in energy homeostasis (*CKB*); suppresses growth and cell arrest (*NDN*); E3 ubiquitin ligase (*RNF41*); lactate dehydrogenase A (*LDHA*) and cell proliferation including myosin in muscle cells (*UNC45A*) (OMIM www.omim/org, accessed on 25 May 2025; Gene Cards www.genecards.org, accessed on 25 May 2025).

#### 3.1.2. Angelman Syndrome

Angelman syndrome is the second recognized chromosome 15q11-q13 genomic imprinting disorder, due to maternal origin, with tissue-specificity of the causative *UBE3A* gene and its expression. The UBE3A (ubiquitin-protein ligase E3A) protein is recognized as E6AP ubiquitin-protein ligase, an enzyme member of the ubiquitin protein degradation system involved in targeting proteins with ubiquitin for degradation within cells. This gene includes ligase activity and is a transcription coactivator or regulator. The E6/E6-AP complex binds to and targets the p53/TP53 tumor-suppressor protein for ubiquitin-mediated proteolysis. Cellular proteasomes recognize and digest ubiquitin-tagged proteins as a normal process to remove damaged or unnecessary proteins to maintain normal cellular function, particularly playing a critical role in the development, morphogenesis, and functioning of the nervous system. This includes synapses for cell-to-cell communication and plasticity. Synaptic plasticity is critical for learning and memory. *UBE3A* also plays a role in hormone regulation, particularly progesterone and circadian clock function (https://medlineplus.gov, accessed on 25 May 2025; OMIM; https://www.genecards.org, accessed on 25 May 2025).

##### Key Imprinted Gene in Angelman Syndrome

*UBE3A*

UBE3A or ubiquitin-protein ligase E3A is an imprinted gene with an encoded protein recognized as E6AP ubiquitin-protein ligase that targets proteins for degradation within cells and as a transcription coactivator that causes AS. It also binds to p53/TP53 tumor-suppressor protein. UBE3A protein plays a role in hormone regulation and signaling, intracellular trafficking, negative regulation of autophagy, and recycling of endosomes. It is recognized for a role in telomerase activity to maintain chromosomal telomeres and stability, circadian function, and is important for the morphogenesis of the nervous system, including synapse communication and plasticity to maintain electrochemical gradients, which are critical for learning and memory.

##### Computational Biology in Angelman Syndrome

*UBE3A*

**Figure 5:**
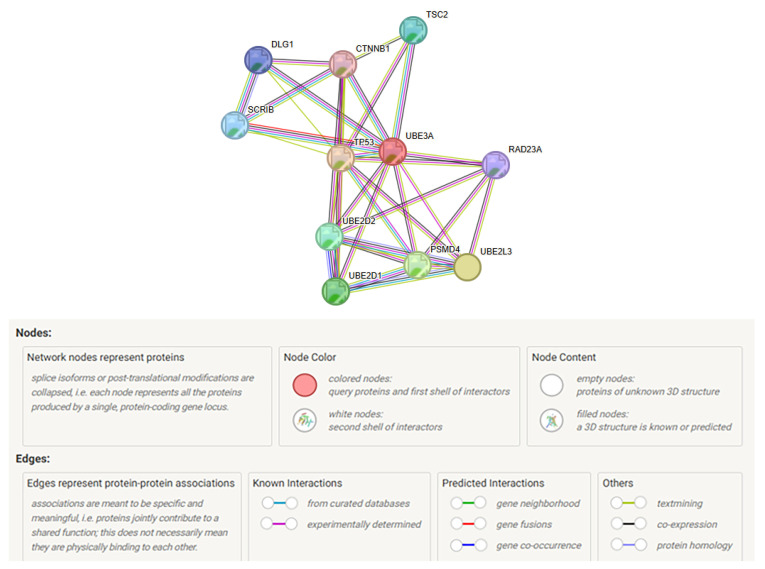
STRING protein–protein interaction network involving the *UBE3A* gene transcript with functional interactions showing 10 protein nodes and UBE3A with 34 protein edges representing different functions. The predicted functional associations included biological processes and molecular functions [71].

The STRING web-based program and database, and other programs were used to study the UBE3A gene in AS, and the top ten significantly associated proteins are illustrated in Figure 5.

The top 10 encoded proteins associated with UBE3A were TSC2, CTNNB1, DLG1, SCRIB, TP53, RAD23A, UBE2D2, UBE2D1, PSMD4, and UBE2L3. The description of predicted protein functions for the 10 associated proteins with UBE3A is listed in Table 6. These 10 interactive proteins or nodes with UBE3A represent all proteins produced, including isoforms for each single protein coding gene, and 34 edges, which indicate both direct predicted functional roles and physical protein associations, processing, or interactions for each gene (see Table 7). The top biological processes identified or interacting with the *UBE3A* gene or protein were protein polyubiquitination, followed by regulation of protein modification by small protein conjugation or removal of misfolded, damaged, or proteins no longer required for cellular function, and for the ubiquitin-dependent protein catabolic process. For STRING biological processes, the ubiquitin-dependent protein catabolic process shared 6 nodes, followed by protein polyubiquitination with 5 nodes. The top molecular functions identified or interacting with the *UBE3A* gene or protein were the ubiquitin conjugating enzyme activity, followed by phosphatase and enzyme binding.

For the STRING website, the predicted categorical functions for the *UBE3A* gene included biological processes with ubiquitin-dependent protein catabolic process, sharing 6 nodes, followed by protein polyubiquitination, with 5 nodes. The top molecular functions identified or interacting with the *UBE3A* gene or protein were ubiquitin conjugating enzyme activity, followed by phosphatase binding and enzyme binding. For the STRING website category of molecular functions, enzyme binding shared 7 nodes, followed by phosphatase binding with 4 nodes, and ubiquitin conjugating enzyme activity with 3 nodes. The top biological process (protein polyubiquitination) and molecular function (ubiquitin conjugating enzyme activity) shared 3 of the 10 nodes studied (UBE2D1, UBE2D2, and UBE2L3) (see Table 6).

##### Biological General Repository for Interaction Datasets (BioGRID)

BioGRID is a separate, searchable web-based program and dataset used for protein–protein functional interactions. The results from this search are shown in Table 8. When searching for UBE3A in AS.

##### Pathway Commons Web-Based Program

**Figure 6:**
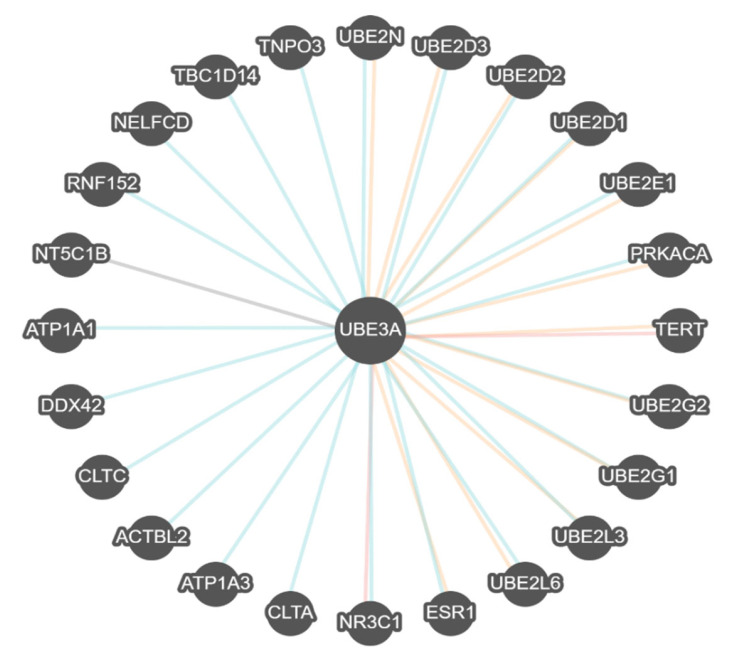
*UBE3A* gene–gene functional interactions represented by binding (blue lines), co-expression (red lines), modification (orange lines) and other (gray lines) for Angelman syndrome.

Pathways Commons is a separate searchable program that focuses on gene–gene regulatory networks, functional interactions, and pathways enriched for related gene expression patterns and binding among interactive or shared processes. Figure 6 shows the gene–gene interactions for the *UBE3A* gene in AS.

In Figure 6, the ***UBE3A*** gene interacts with 24 genes, and nine from the *UBE2* gene family are involved with the modification of proteins with ubiquitin to target abnormal or short-lived proteins for degradation (*UBE2L6*, *UBE2L3*, *UBE2G1*, *UBE2G2*, *UBE2E1*, *UBE2D1*, *UBE2D2*, *UBE2D3*, *UBE2N*). Other genes and encoded proteins identified do impact P-type cation transport ATPases and sodium/potassium types, integral membrane proteins needed to establish and maintain electrochemical gradients (*ATP1A1*, *ATP1A3*). The DEAD box protein family member implicated in cellular processes involving translational initiation, nuclear and mitochondrial splicing, ribosome and spliceosome assembly (*DDX42*); telomerase enzyme maintains chromosomal telomeres (*TERT*) needed for chromosome integrity; catalytic subunit of protein kinase A (*PRKACA*); nuclear import receptor for serine/arginine-rich proteins such as splicing factors (*TNPO3*); protein kinase binding with negative regulation of autophagy and recycling of endosomes (*TBC1D14*); negative elongation factor complex for RNA polymerase II (*NELFCD*); small GTPase binding activity and ubiquitin protein ligase activity involving several processes including cellular response to amino acid starvation, negative regulation of TORC1 signaling and protein ubiquitination (*RNF152*); cytosolic 5-prime nucleosidases to catalyze production of adenosine regulating diverse physiological processes (*NT5C1B*); Clathrin, a protein coat for cellular organelles involved with intracellular trafficking of receptors and endocytosis of several macromolecules (*CLTA*, *CLTC*); estrogen receptor and ligand-activated transcription factor (ESR1); glucocorticoid receptor (*NR3C1*) and protein kinase binding activity impacting postsynaptic actin cytoskeletal involved in axonogenesis and cell motility (*ACTBL2*) (OMIM, www.omim/org, accessed on 25 August 2025) (GeneCards, www.genecards.org, accessed on 25 August 2025).

### 3.2. Laboratory Genetic Testing in Prader–Willi and Angelman Syndromes

#### 3.2.1. Laboratory Methods for Prader–Willi and Angelman Syndromes

Historically, advanced laboratory methods in the early 1980s led to high-resolution chromosome karyotypes and a two-to-three-fold increased level of detection of cytogenetic defects, including the 6 Mb size deletion of the 15q11-q13 region seen in the majority of those with PWS and AS. It was first reported by Ledbetter and others in 1981 [76]. Later, Butler and Palmer in 1983 [77] used chromosome 15 polymorphic staining and found the chromosome 15q11-q13 deletion was de novo and of paternal origin. In 1989, the discovery of DNA markers identified in the 15q11-q13 region was used by Nicholls, Butler, and others [78] to demonstrate that individuals with PWS with normal appearing high-resolution chromosome 15s showed both 15s from the mother or maternal disomy 15. These observations led to the concept of genomic imprinting, with later observation of the same deletion in AS but of maternal origin.

Continued development of commercially available DNA probes and fluorescent in situ hybridization (FISH) in the early 1990s resulted in discoveries of microdeletions that were not detectable with high-resolution chromosome analysis and dozens of microdeletion syndromes identified. Methods using DNA Southern blotting, polymorphic DNA microsatellites, DNA methylation patterns, and fluorescence in situ hybridization (FISH) were applied to detect chromosome 15q11-q13 deletions and parent of origin, such as uniparental disomy 15, in both PWS and AS and in other disorders. In the 2000s, chromosome microarrays were developed, and now, with ultra-high resolution arrays using millions of DNA polymorphic probes that can detect subtle chromosome defects at the gene level with resolution far beyond what was available in the 1980s or 1990s. These include microdeletions, microduplications, or loss of heterozygosity (LOH), which are needed to identify uniparental disomy 15 subclasses (those with greater than 8 Mb in size) and imprinting center defects. For example, the presence of maternal disomy 15 with specific subclasses (total isodisomy or segmental) can impact the diagnosis, medical care, surveillance, and genetic counseling, as a second genetic disorder may result in an individual with PWS with maternal disomy 15 due to the mother being a carrier of a recessive gene allele defect located in the LOH region. The PWS child could then have two identical copies of the mother’s defective recessive allele. There are potentially hundreds of disease–causing genes found on chromosome 15 [14,15,16,79]. The advent of DNA sequencing and advanced genomic technology with computer-based analysis has revolutionized the study of rare genetic disorders and single gene mutations, including *UBE3A* gene variants and defects, as the second most common cause in AS [5]. Single gene variants (e.g., *SNRPN*) causing PWS are quite rare [14,74].

Several laboratory methods have been utilized to detect errors for genomic imprinting, including methylation-specific multiple-ligation probe amplification (MS-MLPA) with DNA markers from the 15q11-q13 region. This assay can detect DNA methylation errors, deletions, imprinting center microdeletions, and uniparental disomy 15. Ultra-high-resolution DNA arrays utilizing millions of probes can identify 80% of the defects seen in PWS. Additionally, digital droplet PCR was developed in the past ten years to identify small deletions, including the imprinting center and low-level mosaicism. Whole exome or genome DNA sequencing can be helpful, as well, in detecting deletions/duplications (del/dup) important for genetic counseling [11,14,80,81].

**Figure 7:**
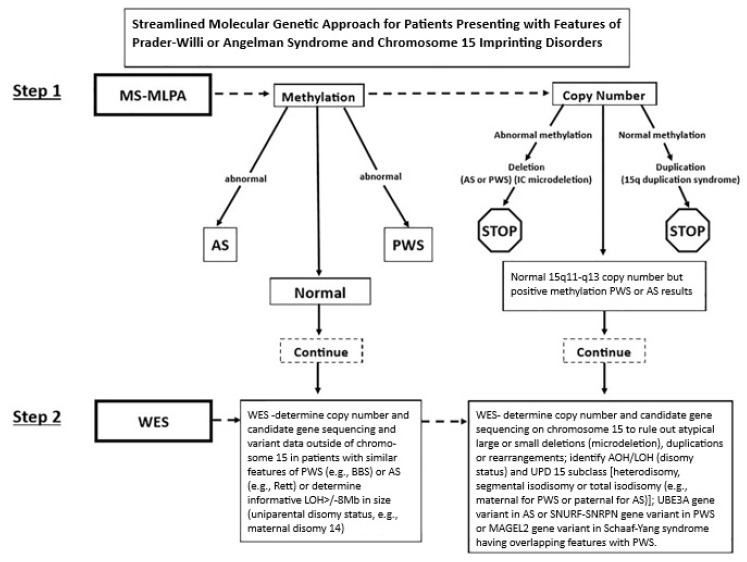
Prader–Willi and Angelman syndromes streamlined molecular analysis and laboratory workflow. Step 1 is the methylation-sensitive MLPA (MS-MLPA) assay for chromosome 15 for methylation status and copy number assignment of the 15q11-q13 region, specifically the imprinting center. Based on the results of Step 1, then proceed to Step 2 with whole-exome sequencing (WES) for determining copy number and sequencing of genes on chromosome 15 and elsewhere to determine heterozygosity status for AOH and/or LOH; UPD, uniparental disomy; PWS, Prader–Willi syndrome; AS, Angelman syndrome; AOH, absence of heterozygosity; IC, chromosome 15q11 imprinting center; and LOH, loss of heterozygosity. Modified and described from Strom et al. [82] in 2021 with permission. The dashed arrows represent testing options for Step 1 and Step 2 depending on test results. The solid line rectangles, boxes or octagons represent a definitive test result while the dashed line rectangles represent more required testing.

A streamlined approach for PWS and AS molecular diagnostics was described by Strom, Butler, and others in 2021 [82], utilizing a combination of modified exome DNA sequencing protocol, del/dup analysis, gene variants, loss of heterozygosity, and DNA methylation with MS-MLPA utilizing chromosome 15 probes to identify PWS and AS molecular genetic classes with imprinting center microdeletions. A streamlined genetic testing flow chart is shown in Figure 7 for screening patients with features of PWS or AS and those disorders with overlapping features such as obesity-related genetic disorders [11,27,83]. Similarly, this approach can be useful in screening related disorders with features of Angelman syndrome, including *MECP2* gene defects in Rett syndrome, which is helpful for the clinician and family members.

#### 3.2.2. Genetic Testing Results

##### Prader–Willi Syndrome

The largest PWS cohort recruited consecutively [84] was studied by Butler and others in 2023 [79] to further characterize genetic defects in males and females, aged 4 years and older. High-resolution chromosomal microarrays were performed on 154 individuals, and 87 (57%) showed the typical 15q11-q13 deletion, with 35 (23%) having the larger typical 15q11-q13 Type I deletion at breakpoints BP1 and BP3, and 52 (34%) with the smaller typical Type II deletion at BP2 and BP3. One individual had the Type I deletion, but also a small duplication of 15q13.3 (433 kb in size) containing two genes (*OTUD7A* and *CHRNA7*). Of those with the Type II deletion, one individual had a small duplication at 15q13.1-q13.2 (187 kb in size) containing two under-characterized genes (*TJP1* and *GOLGA8*) located between breakpoints BP4 and BP5. Atypically smaller or larger deletions between breakpoints BP1 or BP5 were found in five individuals. Sixty-two individuals (40%) showed maternal disomy 15 with segmental isodisomy 15 in 34 subjects (22 females, 12 males), or 55% and 15q26.3, 15q14, and 15q26.1 bands most involved, but isodisomic regions were noted throughout chromosome 15. Segmental isodisomy 15 is due to normal crossover events in maternal female meiosis I with normal female chromosome 15 segregation. The average size of the total loss of heterozygosity (LOH) was 27.1 Mb in isodisomic regions, and each LOH varied in size from 5.6 to 52.2 Mb with an average of 18.4 Mb. One male PWS subject had segmental isodisomy 15, but also showed an extra X chromosome consistent with an XXY male pattern or Klinefelter syndrome, along with segmental isodisomy X. Both X and 15 chromosomes were of maternal origin. Twenty-four (39%) of the 62 individuals with PWS showed microarray patterns indicating maternal heterodisomy 15, non-deletion subclass, or a rare non-deletion (epimutation) imprinting center defect, requiring parental DNA testing to confirm the parent of origin (both 15s from the mother supporting maternal heterodisomy, or biparental inheritance supporting an epimutation). Maternal heterodisomy 15 arises when no cross-over events occur in female meiosis I. Total isodisomy 15 was found in four individuals with PWS and represents an LOH of the entire long arm of chromosome 15 due to errors in female meiosis II [79].

##### Angelman Syndrome

Four recognized AS molecular genetic classes are categorized by the impact on methylation of the chromosome 15 region; all with disturbances of the imprinted maternally expressed *UBE3A* gene. The *UBE3A* gene is expressed in the brain from maternal chromosome 15 only. These defects include the typical 15q11-q13 deletion of maternal origin, paternal disomy 15, *UBE3A* gene mutations, and imprinting center defects. The most common is a deletion of the 15q11-q13 region, as similarly seen in PWS, but of maternal origin in about 75% of AS patients [62]. As in PWS, two typical de novo 15q11-q13 deletions (larger Class or Type I and smaller Class or Type II) are found in AS. The typical 15q11-q13 Type I deletion is found in about 40% of those with AS. Atypical deletions may be larger or smaller than normal or between the proximal breakpoint BP1 and the distal breakpoint BP5. Unbalanced chromosomal 15 translocations or inversions, which rearrange genetic material, may increase in the recurrence risk for additional children with AS, but are rarely present.

Paternal disomy 15 accounts for about 5% of individuals, while *UBE3A* gene mutations are found in 11% of AS cases [8,61]. *UBE3A* gene mutations, if present, could be maternally inherited, and targeted testing in the mother and the patient’s maternal grandfather may be warranted to assess more accurate recurrence for other family members. A 50% recurrence risk in her future offspring may be accurate if deemed inherited [11]. About 3% of individuals with AS have an imprinting center defect, which impacts the expression of imprinted genes, specifically *UBE3A*, as the epigenetic marking fails to properly switch in the germline. Mosaic cases of imprinting center defects have been identified, and a percentage of cells lack gene expression in the 15q11-q13 region. This phenomenon may be more common than previously thought [11,20]. Mosaicism can also complicate genomic testing and interpretation of results, requiring more research and testing of these rare genetic disorders [20,85,86,87].

Recent genetic laboratory approaches are useful for identifying PWS and AS [82] and can test for genes causing overlapping features with PWS (e.g., obesity related genetic disorders, including Alstrom, Bardet–Beidel, Cohen, Temple, fragile X [11,15,27,83], obesity genes such as *LEPR*, *BDNF*, *FTO*, *SH2B1*, *POMC*, *MCR4*, *TUB*, *SNRPN*, *MAGEL2,* and *AGRP* [54], or with AS (e.g., *MECP2* and Rett syndrome) and *UBE3A*. The streamlined approach can identify 97% of cases with PWS and presumably for AS (83) (see Figure 7). Other disorders identified could include 15q duplications, microdeletions (e.g., 22q11.2, 16p11-p13, Williams, Smith–Magenis), and other imprinting syndromes (such as Russell–Silver, GNAS-related disorders, and Beckwith–Weidemann) [11,15,82]. An accurate diagnosis in both PWS and AS is required for treatment, medical care, surveillance, and genetic counseling.

#### 3.2.3. Other Genetic Testing Approaches

Long-read DNA sequencing for the detection and subtyping of PWS and AS genetic defects is now under investigation, including genome-wide nanopore sequencing for molecular diagnosis. Adaptive Oxford Nanopore Technologies (ONT) long-reads have shown the possibility to examine methylation and underlying pathogenic mechanisms using a single test approach for clinical interpretation and diagnosis [20,88,89,90,91]. ONT technology may also detect methylation patterns applicable in other disorders, particularly neurodevelopmental disorders, in addition to AS and PWS [86]. Methylation mosaicism in the 15q11-q13 region has also been reported in PWS and AS with atypical clinical findings [85,92]. For example, individuals with AS and mosaicism are reported to have higher intellectual functioning than those with typical AS, while individuals with PWS and methylation mosaicism due to maternal disomy 15 with mosaic trisomy 15 are found without hypotonia at birth [93].

Genotype–phenotype observations have emerged in rare diseases, including PWS and AS, having different genetic subtypes or subclasses, but there is a lack of peripheral tissue to use, including blood-based biomarkers for prognostic application, co-occurring conditions, especially psychiatric illness, and hyperphagia, with a lack of understanding of underlying mechanisms, some of which are addressed in this review. To address cell-type-specific differential expression patterns in the brain and other tissues from genes in and outside the 15q11-q13 region, Shahrokhi et al. [94] reported transcriptomic signatures related to cognitive and psychiatric phenotypes in PWS and defined gene expression changes across different cell types in the prefrontal cortex (PFC) compared to controls. Relationships and changes in both blood and brain with PWS symptoms were studied using long non-coding RNAs and protein-coding genes and single-nucleus RNA sequencing (snRNA-seq). They showed an increased proportion of inter-neurons in both PWS deletion and non-deletion groups compared with controls, and 54 genes showed related pathways consistently dysregulated across all cell types found in the PFC of the PWS groups compared with controls. Specifically, RPS18, a ribosomal protein component of the 40S subunit, is involved in the initiation of translation, with pathways involved in peptide chain elongation and nervous system development. It was the only protein-coding gene upregulated in the PWS PFC across all comparisons. Increased *RPS18* mRNA levels were also found in peripheral blood mononuclear cells and associated with intellectual functioning and challenging behaviors, but not autistic traits, in PWS children due to the non-deletion status. This study would further suggest the development of prognostic biomarkers impacting therapeutics targeting dysregulated genes and related pathways between the brain and the periphery. Furthermore, in silico approaches for drug repurposing using gene expression and proteomics data from the brain for targetable disturbed pathways in rare genetic disorders will be essential in future investigations. In addition, the relationships between *UBE3A* and other expression patterns using novel normalized droplet digital polymerase chain reactions were analyzed in peripheral blood cells in controls, PWS, and AS subjects, and features of autism and developmental functions were recorded [95]. All developmental functioning scores were positively correlated with *UBE3A* expression in participants with AS having the 15q11-q13 deletion and autism features in the non-deleted PWS group, indicating novel interactions between specific gene expressions in blood and brain processes underlying motor and language impairments and autism.

### 3.3. Clinical Trial Experiences in Prader–Willi and Angelman Syndromes

Recent clinical trial studies have shown promise in treating both PWS and AS [96,97], as standard dietary restriction, exercise, and growth hormone replacement in PWS have been suboptimal. The treatments include beloranib, setmelanotide, diazoxide, choline extended-release tablet (DCCR), an unacylated ghrelin analog, oxytocin and related compounds, brain histamines, bitter taste receptors, glucagon-like peptide 1 receptor agonist, bariatric surgery, vagal nerve, and transcranial direct-current stimulation [96,97,98,99,100,101,102]. To date, ongoing or completed trials have led to limited or no success for hyperphagia, but DCCR has recently received FDA approval [103]. Other features in PWS, such as daytime sleepiness or anxiousness using intranasal carbetocin, an oxytocin analog, have been studied [104]. Other medications considered are lorcaserin, naltrexone HCl/bupropion HCl, and phentermine/topiramate [105].

Loss of expression of the maternal *UBE3A* gene in AS occurs via several molecular mechanisms, including deletions or frameshift, missense or nonsense mutations. UBE3A plays a critical role in activity-dependent synaptic plasticity during neurodevelopment, leading to abnormalities of the dendritic spine when disturbed, impaired long-term potentiation, and an ataxic phenotype [106]. The paternal copy is silenced by a long non-coding antisense transcript, termed *UBE3A-ATS*, which has been studied in both mice and humans [106]. This gene silencing takes place on the paternal allele on chromosome 15, where the transcription of the *UBE3A-ATS* is regulated from areas upstream to the Prader–Willi syndrome imprinting center (PWS-IC) and runs through the *SNURF*/*SNRPN* region, *SNORD116*, *IPW*, *SNORD115,* and to the *UBE3A* coding region in an antisense orientation [107]. The use of agents that silence the paternal *UBE3A-ATS* expression may lead to potential treatment in Angelman syndrome. These include antisense oligonucleotides (ASOs) that are complementary to the distal part of the *UBE3A-ATS* and thereby potentially increase paternal *UBE3A* expression. Two ASO experiments under investigation, namely GTX-102 and RO7248824, and a third, ION582, are under development [106,108,109]. GTX-102 showed promise when administered intrathecally in five patients with AS and improved scores in clinical global impression and both receptive and expressive communication, but all five patients with AS presented with adverse events, including lower limb weakness, receiving the highest doses. Lower limb weakness did resolve with time. These findings may be related to inflammation of the meninges. Another promising therapeutic approach for AS is the use of topoisomerase inhibitors in both in vitro and in vivo experiments [110,111,112,113]. Furthermore, genome engineering approaches in mice include CRISPR/Cas9 technology [114,115,116]. Other therapeutic strategies in the AS pipeline are those that aim to restore the missing or non-functional UBE3A protein in the neurons via gene replacement or enzyme therapies [116,117,118]. An adeno-associated virus-mediated gene replacement therapy approach is also under development [106].

## 4. Conclusions, Limitations, and Future Directions

A review of the clinical and genetic findings in both Prader–Willi and Angelman syndromes, along with searchable web-based integrated genetic approaches, is described with natural history and impact of genetic defects on severity and clinical presentations. Health care management and guidance with clinical trial experiences have been reported for both PWS [14,26,83,119,120] and AS [116,121,122].

PWS and AS were the first examples of errors in genomic imprinting due to differences in parent of origin impacting clinical presentations. They do share common molecular genetic classes, which are clinically distinct from the presentations described and reviewed. Paternal 15q11-q13 deletions cause PWS with several imprinted genes, specifically *SNRPN* and *MAGEL2*, and maternal deletion of the same chromosome region causes AS due to errors of one imprinted gene (*UBE3A*). Past and present genetic laboratory approaches and results were summarized and illustrated with advances in testing, limitations, and future directions discussed.

Different neurodevelopmental-behavior and clinical findings are noted in both disorders, but with more cognitive deficits in AS, including lack of speech, abnormal gait, and seizures. They may not present for evaluation until later in infancy with the onset of seizures, while those with PWS have severe infantile hypotonia, lack of suck, and hypogonadism/hypogenitalism with cryptorchidism at birth and hyperphagia in childhood [16,20,25,26,123]. Neither disorder presents congenital anomalies or internal organ malformations but shares neurological deficits, abnormal brain function, and central nervous system development. Our review and analysis of existing data summarized clinical findings and genetics undertaken using an integrated genetic analysis of three web-based programs to identify existing predicted protein–protein or gene–gene interactions, molecular mechanisms, pathways, biological processes, and disease associations of causative genes for PWS and AS.

*SNRPN* encodes an important polypeptide of the small nuclear ribonucleoprotein complex required for pre-mRNA processing, spliceosome formation, and tissue-specific alternative splicing that impacts production of several interrelated proteins impacting several clinical findings in PWS. These include regulation of hormones, reproduction, circadian rhythm, behavior, and appetite. The promoter region overlaps with the imprinting center necessary for DNA methylation and gene activity. The *MAGEL2* gene interacts with multiple genes, both imprinted and non-imprinted, and is a member of the melanoma antigen gene family with enhancement of ubiquitin ligase activity and protein degradation, reproduction, sexual development, appetite, hormone control, and circadian rhythm. Defects in this gene also cause a separate disorder (Schaaf–Yang syndrome) with overlapping features in PWS. Ubiquitin ligase is highly related to the *UBE3A* gene in AS.

Computational analysis of existing data showed that *MAGEL2* interacts with the *TRIM27* gene involved with transcriptional repressor activity and interacts with OXT (oxytocin), a hormone related to basic human behavior and reproduction used in clinical trials [105]. Interestingly, fewer oxytocin neurons are reported in the hypothalamus of PWS [124], supported by abnormal microarray gene expression patterns [125]. Our study also noted a direct relationship with the *MAGEL2* gene and both imprinted and maternally expressed *UBE3A* and *ATP10A* genes in the 15q11-13 region, indicating more than a physical connection among the genes in the region. The *UBE3A* gene is tissue-specific, and its encoded protein is a ubiquitin-protein ligase that leads to ubiquitin-mediated proteolysis for the removal of damaged or unnecessary proteins to maintain normal cellular function, critical for nervous system development and function.

Genes in the 15q11-q13 critical region could potentially have epigenetic effects outside the region and account for inter-patient diversity. To further understand individual-specific gene abnormalities, whole genome sequencing may be warranted for both disorders and could lead to a more comprehensive and individualized treatment plan, including disturbed genes and their encoded proteins leading to disease. Furthermore, those with PWS and maternal disomy 15, specifically isodisomic subclasses involving chromosome 15 regions, if the mother is a heterozygous carrier of an autosomal recessive disorder (e.g., Tay–Sachs) [23,79].

A central limitation of a review is the use of the existing literature and analysis based on searching websites and tools. We cannot verify or repeat experiments or re-evaluate existing or new patients with rare diseases at this point to address potential new clinical or genetic findings. We can only propose new evaluations and laboratory studies based on gaps in knowledge identified from the review of known causes and disease mechanisms summarized from existing information. Additionally, individualized pharmacogenetics for medication management [126] should be further studied. Specific treatment options in PWS or AS could also be developed using individualized treatment plans, as seen for cystic fibrosis [127,128]. Cystic fibrosis is an autosomal recessive condition caused by mutations in a single gene (*CFTR*), but with phenotypic heterogeneity related to the type of mutation found per patient and response to drug (e.g., Phe508del *CFTR* gene defect only) [128]. The concept of thera-typing, that is, matching specific drugs to genetic defects, may apply to PWS and AS, as well, based on the biological processes, pathways, and molecular mechanisms reviewed in this report. Important information to assess treatment based on gene defects within and outside of the 15q11-q13 region should be further characterized. Lastly, rare genetic disorders such as PWS and AS present with clinical barriers and limitations for treatment discovery due to genetic heterogeneity, understanding of disease mechanisms, inadequate natural history, and a sparse pool of recruitable subjects with funding for clinical trial studies [129]. Our observations may stimulate more investigations to better understand the complexity of this specific imprinted region and the joint connection between PWS and AS. Furthermore, computational observations in the future may open new avenues for research, drug development, and treatment needed for both disorders.

Basic and advanced research on the genetics or treatment of these imprinted disorders will continue to be undertaken, including single-cell RNA sequencing of brain and non-brain tissue with whole genome sequencing from PWS, AS, and controls to characterize imprinted and non-imprinted gene expression patterns and relationships with proteomics and drug rediscovery. More effort is needed to study therapeutic options, description, and treatment of comorbidities impacted by genetic imprinting, genomics, and epigenetics in both disorders. More laboratory research on brain samples collected for research, stem cell, and organoid development should be encouraged with DNA and RNA sequencing and proteomics. More organized natural history studies and clinical trials should be performed with objective and improved measures to monitor results that impact care and quality of life in both PWS and AS. Monitoring the change in care with age, gender, syndrome-related treatment such as growth and other hormones, and genetic subtypes will be important, as well as the use of pharmacogenetics and response to medications in rare genetic disorders [126,130].

Medical management for both PWS and AS should be improved beginning in infancy, requiring more awareness and an earlier diagnosis to address their clinical presentations and genetic findings with molecular genetic class-phenotypic differences. Both disorders require a multidisciplinary team approach to include clinical geneticists, primary care physicians, orthopedic specialists, occupational (OT), physical (PT) and speech-language therapists (SLP), mental health experts (psychologists, psychiatrists), developmental pediatrician input, sleep specialists, endocrinologists and continuous input by dietitians to monitor food sources, caloric input and weight gain, specifically for PWS, throughout life [14]. A team of experts to treat AS includes clinical geneticists, neurologists for seizure evaluation and management, specialized therapists to include PT, OT, and SLP services, sleep specialists, gastroenterology, physical medicine, rehabilitation, and orthopedics specialists, and mental health experts [116]. An appropriate medical care management team and counselors for PWS are needed to monitor increased weight gain, diet restrictions, and exercise programs, monitoring and assessments for associated comorbidities, including self-injury, and for other behavioral and psychiatric problems, including self-injury, aberrant behavior and educational concerns. Growth and other hormone deficiencies in PWS will be addressed and treated along with rigorous control of diet, food intake, and food security. A patient with AS will require early intervention to include input for specialized therapeutics, including augmentative, and the use of assistive communication devices for development and cognitive skills. Early signs of seizures and neurodevelopmental needs should be monitored in AS. Early diagnosis with confirmation of the PWS or AS molecular genetic classes is essential to ensure adequate intervention, treatment, and genetic counseling of family members for both PWS and AS and to improve quality of life.

## Figures and Tables

**Table 1:** Description of predicted functions of the top ten associated proteins with SNRPN *.

*Protein Symbol*	*Description*
**SNRPD1,** **SNRPD2,** **SNRPD3**	Small nuclear ribonucleoproteins Sm D1, Sm D2, and Sm D3 play roles in pre-mRNA splicing as a core component of the SMN-Sm complex that mediates spliceosomal snRNP assembly and are components of the spliceosomal U1, U2, U4, and U5 small nuclear ribonucleoproteins (snRNPs), the building blocks of the spliceosome.
**SNRPA,** **SNRPC,** **SNRPE,** **SNRPF,** **SNRPG**	Small nuclear ribonucleoproteins A, C, E, F, and G play roles in pre-mRNA splicing as a core component of the SMN-Sm complex that mediates spliceosomal snRNP assembly, and also components of the spliceosomal U1, U2, U4, and U5 small nuclear ribonucleoproteins (snRNPs).
**SNRNP70**	U1 small nuclear ribonucleoprotein 70k Da, a component of the spliceosomal U1 snRNP, is essential for recognition of the pre-mRNA 5′ splice-site and subsequent assembly of the spliceosome that binds to the loop I region of U1-snRNA.
**LSM4**	U6 snRNA-associated Sm-like protein LSm4 plays a role in pre-mRNA splicing as a component of the U4/U6-U5 tri-snRNP complex involved in spliceosome assembly and part of the precatalytic spliceosome B complex.

* STRING database (www.string-db.org, accessed on 25 May 2025); gene cards database (www.genecards.org, accessed on 25 May 2025).

**Table 2:** STRING: Predicted classification and functional analysis of the *SNRPN* gene *.

*Biological Process (Gene Ontology)*	*Molecular* *Function (Gene Ontology)*	*Cellular Component (Gene Ontology)*	*KEGG Pathways*	*Reactome Pathways*	*Annotated* *Keywords*
Spliceosomal snRNP assembly	snRNA binding	U1 snRNP	Spliceosome	mRNA splicing-major pathway	Ribonucleoprotein
7-methylguanosine cap hypermethylation	U1 snRNP binding	Spliceosomal snRNP complex		SARS-CoV-2 modules host translation machinery	Spliceosome
U2-type prespliceosome assembly	U1 snRNA binding	U4/U6 x U5 tri-snRNP complex		mRNA splicing-minor pathway	mRNA processing
mRNA splicing via the spliceosome	RNA binding	U4 snRNP		snRNP assembly	RNA-binding
Spliceosomal complex assembly		U2-type spliceosomal complex		SLBP independent processing of histone pre-mRNAs	Nucleus

* STRING database (www.string-db.org, accessed on 25 May 2025) [71]. The STRING-predicted function data are listed with the most significant value based on count in the network, strength, signal, and FDR or false discovery rate as described in the Review of Data Strategy section and found first in each column. For example, the *p*-value for biological process (Spliceosomal snRNP assembly) was 3.59 × 10^−16^; for molecular function (snRNA binding) was 1.18 × 10^−7^; for cellular component (U1 snRNP) was 1.85 × 10^−25^; for KEGG pathway (Spliceosome) was 1.0 × 10^−18^; for reactome pathway (mRNA splicing-major pathway) was 4.36 × 10^−19^; and for annotated keywords (Ribonucleoprotein) was 3.74 × 10^−18^. All other FDR-derived *p*-values for features in each column or category were significant at *p*-values less than 0.05.

**Table 3:** Description of predicted functions of the top ten proteins associated with MAGEL2 *.

*Protein Symbol*	*Description*
**TRIM27**	TRIM27 Zinc finger protein acts as an E3 ubiquitin-protein ligase that mediates ubiquitination of PIK3C2B and inhibits its activity, including CD4 T-cell activation. It also has transcriptional repressor activity with rhythmic processing.
**USP7**	Ubiquitin carboxyl-terminal hydrolase 7 deubiquitinates target proteins such as FOXO4, p53/TP53, MDM2 and PTEN also enhance the E3 ligase activity for proteasomal degradation, protein recycling, rhythmic processing, and apoptosis.
**MKRN3**	E3 ubiquitin-protein ligase makorin-3 catalyzes the covalent attachment of ubiquitin moieties onto substrate proteins.
**SNRPN**	Small nuclear ribonucleoprotein-associated protein N shows potential involvement in tissue-specific alternative RNA processing events.
**ATP10A**	Phospholipid-transporting ATPase acts as a cation transport ATPase family that catalyzes the hydrolysis of ATP coupled to the transport of amino phospholipids from the outer to the inner membranes implicated in vesicle formation and uptake of lipid signaling molecules.
**OXT**	Oxytocin-neurophysin 1 is a peptide hormone produced by the hypothalamus and affects aspects of human behavior, including attraction, bonding, and the male and female reproductive systems.
**CSAG1**	Chondrosarcoma-associated gene 1
**UBE3A**	Ubiquitin-protein ligase E3A accepts ubiquitin from an E2 ubiquitin-conjugating enzyme in the form of a thioester and transfers it to several substrates, which are involved in DNA replication. It functions as a cellular quality control and helps in the degradation of cytoplasmic misfolded proteins.
**NPAP1**	Nuclear pore-associated protein 1 shows involvement in spermatogenesis.
**VPS35**	Vacuolar protein sorting-associated protein 35 acts as a component of the cargo-selective complex to prevent mis-sorting of selected transmembrane cargo proteins into the lysosomal degradation pathway.

* STRING database (www.string-db.org, accessed on 25 May 2025); Gene Cards database (www.genecards.org, accessed 25 May 2025).

**Table 4:** STRING: Predicted classification and functional analysis of *MAGEL2* gene *.

*Local Network Cluster*	*Wiki Pathways*	*Human Phenotype*	*Annotated Keywords*	*Disease–Gene Association*
		Early onset of sexual maturation		Prader–Willi syndrome
Mostly uncharacterized, including Temple syndrome and Prader–Willi syndrome	Prader–Willi syndrome	Premature pubarche	Biological rhythms	
		Poor suck		Schaaf–Yang syndrome
Prader–Willi syndrome and dyscalculia	Angelman syndrome	Central sleep apnea	Ubl conjugation pathway	
		Polyphagia		Angelman syndrome

* STRING database (www.string-db.org, accessed on 25 January 2025) [71]. STRING-predicted function data are listed with the most significant value based on count in the network, strength, signal, and FDR or false discovery rate as described in the Review of Data Strategy section and found first in each column. For example, the *p*-value for Local Network Cluster (Mostly uncharacterized, …) was 5.56 × 10^−8^; for WikiPathways (PWS and AS) was 1.2 × 10^−12^; for Human Phenotype (Early onset of sexual maturation) was 7.92 × 10^−5^; for Annotated Keywords (Biological rhythms) was 2.2 × 10^−2^; and for Disease–gene Association (PWS) was 7.94 × 10^−8^. All other FDR-derived *p*-values for features in each column or category were significant at *p*-values less than 0.05.

**Table 5:** Gene ontology biological processes, molecular functions, and cellular components using BioGRID protein–protein functional interaction for SNRPN and MAGEL2 in Prader–Willi syndrome.

Biological Processes		Molecular Functions		Cellular Components	
SNRPN	MAGEL2	SNRPN	MAGEL2	SNRPN	MAGEL2
RNA Splicing	Arp2/3 Complex-Mediated Actin Nucleation	Protein Binding	Protein Binding	Small Nuclear Ribonucleoprotein Complex	Endosome
	Negative Regulation of Transcription, DNA-Templated		Ubiquitin-Protein Transferase Activity	Spliceosomal Complex	Nucleus
	Protein K63-Linked Ubiquitination				Retromer Complex
	Regulation of Circadian Rhythm				
	Retrograde Transport, Endosome to Golgi				

**Table 6:** Description of predicted functions of the top ten associated proteins with UBE3A *.

*Protein Symbol*	*Description*
**TP53**	Cellular tumor antigen p53 acts as a tumor suppressor in many tumor types, inducing growth arrest or apoptosis depending on physiological circumstances and cell type, with negative regulation of cell division.
**UBE2L3**	Ubiquitin conjugating enzyme E2 L3
**UBE2D1**	Ubiquitin-conjugating enzyme E2 D1 mediates the degradation of selected, short-lived, and abnormal proteins interacting with TP53.
**UBE2D2**	Ubiquitin-conjugating enzyme E2 D2 mediates the degradation of selected, short-lived, and abnormal proteins interacting with TP53.
**DLG1**	Disks large homolog 1 is an essential multidomain scaffolding protein required for normal development and function, including regulation of cardiac myocytes by modulating the functional expression of Kv4 channels.
**TSC2**	Tuberin, in complex with TSC1, acts as a tumor suppressor, inhibiting nutrient-mediated or growth factor-stimulated phosphorylation of S6K1 and EIF4EBP1 by negatively regulating mTORC1 signaling and may play a role in microtubule -mediated protein transport,
**SCRIB**	Protein scribble homolog or a scaffold protein involved in different aspects of polarized cell differentiation, including the regulation of epithelial and neuronal morphogenesis as well as cell proliferation with progression of G1 to S phase. It may play a role in cell adhesion and exocytosis by targeting synaptic vesicles,
**RAD23A**	UV excision repair protein RAD23 homolog A is a multiubiquitin chain receptor involved in the modulation of proteasomal degradation and may bind simultaneously to the 26S proteasome to deliver ubiquitinated proteins to the proteasome.
**CTNNB1**	Catenin beta-1 is a downstream component of the canonical Wnt signaling pathway and is involved in transcription and regulation of cell adhesion.
**PSMD4**	26S proteasome non-ATPase regulatory subunit 4 acts to degrade ubiquitinated proteins, playing a key role in maintenance of protein homeostasis by eliminating misfolded or damaged proteins no longer required for cellular function.

* STRING database (www.string-db.org, accessed on 25 August 2025); gene cards database (www.genecards.org, accessed on 25 August 2025).

**Table 7:** STRING: Predicted classification and functional analysis of the *UBE3A* gene *.

*Biological Process (Gene Ontology)*	*Molecular* *Function (Gene Ontology)*	*Cellular Component (Gene Ontology)*	*KEGG Pathways*	*Reactome Pathways*	*Disease–Gene* *Association*
Protein polyubiquitination		Scrib-APC-beta-catenin complex	Human papillomavirus infection		
Ubiquitin-dependent protein	Ubiquitin conjugating enzyme activity	Myelin sheath abaxonal region	Viral carcinogenesis	Transcriptional regulation by VENTX	Colon carcinoma
catabolic process		Proteasome complex	Hippo signaling pathway	TICAM, RIP1-mediated IKK complex recruitment	
Negative regulation of mitophagy	Phosphatase binding	Cell–cell contact zone	Ubiquitin-mediated proteolysis	IKK complex recruitment mediated by RIP1	Nephroblastoma
Protein K48-linked ubiquitination		Intracellular protein-containing complex	Thyroid hormone signaling pathway	Oxygen-dependent proline hydroxylation of hypoxia-inducible factor alpha	
Negative regulation of the mitotic cell cycle	Enzyme binding				Colorectal carcinoma

* STRING database (www.string-db.org); accessed on 25 August 2025) [71]. STRING-predicted function data are listed with the most significant value based on count in the network, strength, signal, and FDR or false discovery rate as described in the Review of Data Strategy section and found first in each column. For example, the *p*-value for biological processes (protein polyubiquitination) was 2.2 × 10^−4^; for molecular functions (ubiquitin conjugating enzyme activity) was 4.9 × 10^−3^; for cellular components (scrib-APC-beta-catenin complex) was 1.7 × 10^−3^; for KEGG pathways (human papilloma virus infection) was 3.05 × 10^−6^; for reactome pathways (transcriptional regulation by VENTX) was 3.3 × 10^−3^; and for disease–gene associations (colon carcinoma) was 4.67 × 10^−2^. All other FDR-derived *p*-values for features in each column or category were significant at *p*-values less than 0.05.

**Table 8:** Gene ontology biological processes, molecular functions, and cellular components using bioGRID protein–protein functional interaction for UBE3A in Angelman syndrome.

Biological Processes	Molecular Functions	Cellular Components
UBE3A	UBE3A	UBE3A
Androgen Receptor Signaling Pathway	Protein Binding	Cytoplasm
Brain Development	Ubiquitin-Protein Transferase Activity	Nuclease
Protein K48-Linked Ubiquitination		
Protein Autoubiquitination		
Protein Ubiquitination Involved in Ubiquitin-Dependent Protein Catabolic Process		
Proteolysis		
Regulation of Circadian Rhythm		
Regulation of Protein Ubiquitination Involved in Ubiquitin-Dependent Protein Catabolic Process		
Ubiquitin-Dependent Protein Catabolic Process		

## Data Availability

No new data were created or analyzed in this study.
